# Multiple CR Spatiotemporal Compressive Imaging System

**DOI:** 10.3390/s25051334

**Published:** 2025-02-21

**Authors:** Xiaowen Hao, Dingaoyu Zhao, Jun Ke

**Affiliations:** 1 School of Optics and Photonics, Beijing Institute of Technology, Beijing 100081, China; 3120215297@bit.edu.cn (X.H.); zdaybit@outlook.com (D.Z.); 2 Key Laboratory of Photo-Electronic Imaging Technology and System, Ministry of Education of China, Beijing 100081, China; 3 National Key Laboratory on Near-Surface Detection, Beijing 100072, China

**Keywords:** high-speed high-resolution imaging, spatiotemporal compressive imaging, computational imaging

## Abstract

Higher spatial and temporal resolutions are two important performance parameters in an imaging system. However, due to hardware limitations, the two resolutions are usually mutually restricted. To meet this challenge, we propose a spatiotemporal compressive imaging (STCI) system to reconstruct high-spatiotemporal-resolution images from low-resolution measurements. For STCI, we also designed a novel reconstruction network for multiple compression ratio (CR). To verify the effectiveness of our method, we implemented simulation and optical experiments, respectively. The experiment results show that our method can effectively reconstruct high-spatiotemporal-resolution target scenes for nine different CRs. With the maximum spatiotemporal CR of 128:1, our method can achieve a reconstruction accuracy of 28.28 dB.

## 1. Introduction

High-spatiotemporal-resolution imaging finds widespread application in diverse fields, including medicine, security, and commercial settings. However, simultaneously improving both the temporal and spatial resolution of imaging systems presents a significant challenge. High-speed, high-resolution imaging puts forward higher requirements on the hardware performance of the systems. The massive data associated with high-speed, high-resolution image signals, coupled with continuous high-frame-rate acquisition, impose stringent requirements on the data transmission bandwidth of the system and the performance of back-end storage devices [1]. Consequently, in the development of imaging systems, spatial and temporal resolutions always exhibit mutual constraints [2]. Although some high-speed cameras can simultaneously achieve high spatial and temporal resolutions, they frequently encounter limitations in data storage capacity, hindering prolonged operation. Furthermore, the high performance of such detectors typically comes at a considerable cost. Framing cameras, which are capable of extremely high-speed imaging, are essential for capturing transient phenomena. However, their complex structure, high cost, and limited sequence depth have hindered widespread adoption. Compressive imaging (CI) is a computational imaging method based on compressive sensing theory [3]. CI can realize high-resolution imaging that exceeds the limitation of the imaging system by combining back-end reconstruction algorithms [4,5,6]. Based on the spatiotemporal correlations inherent in natural image sequences [7], STCI aims to achieve high-speed and high-resolution imaging of target scenes using CI techniques.It encodes and compresses the target scene in both spatial and temporal dimensions and reconstructs the high-resolution images through the reconstruction algorithm, which effectively alleviates hardware limitations. STCI significantly reduces the amount of data acquired, which alleviates the strain on both the data transmission bandwidth of the system and back-end storage devices, thereby enabling prolonged high-speed, high-resolution imaging. Before our work, there have been some initial studies on STCI. In [8], Treeaporn et al. demonstrated the feasibility and advantages of STCI under high-readout-noise conditions through simulation experiments. The paper also discusses three electro-optical space-time compressive imaging architectures. However, it is difficult to apply to actual imaging systems because of the high complexity of the system. In [9], Harmany et al. combined the coded aperture and the keyed exposure technique to improve spatiotemporal resolution and analyzed the mathematical model. However, the encoding matrix designed using the coded aperture is complex and computationally intensive. In addition, a feasible optical structure is not given for the imaging idea. Ke et al. [10] combined block-wise compressive imaging (BCI) [11] and temporal compressive imaging (TCI) techniques for high-spatiotemporal-resolution imaging. A two-step reconstruction strategy is adopted for reconstruction. However, the quality of the reconstructions is limited. In [12], Tsai et al. discussed CI in the spectral and temporal domains, which presents excellent performance. With advances in light field modulation technology, Zhang et al. proposed end-to-end snapshot compressive super-resolution imaging with deep optics, which adopted the diffraction optical element (DoE) and the TCI technique to realize the improvement of spatial and temporal resolutions [13]. The researchers also proposed an End-to-End network to optimize the DoE and the reconstruction network jointly. The system achieved 128 times data expansion in spatial and temporal domains. The introduction of the DoE element realizes the spatial encoding for target scenes but also increases the complexity of the system.

Reconstruction algorithms play an important role in CI. In previous research, CI reconstruction algorithms have been well studied, consisting mainly of the traditional model-based reconstruction algorithm [14,15,16] and the deep learning (DL)-based reconstruction algorithm [17,18]. For STCI, it encodes and compresses the original data in both temporal and spatial dimensions. Successive image signals exhibit strong spatiotemporal correlations. Therefore, the utilization of spatiotemporal correlations is of significant importance for the design of the STCI algorithm. Furthermore, we found that high-speed motion scenes exhibit more pronounced sparsity in the temporal domain than in the spatial domain. Based on this observation, we discuss a new STCI model and design a novel reconstruction algorithm based on the unfolding network from the point of view of encoding strategy design. In addition, we notice that most reconstruction algorithms are designed for fixed CR. However, for different imaging tasks, such as object detection and recognition, the requirements for imaging quality or resolutions vary depending on the scene and task requirements. Adaptively adjusting the CR based on the imaging task and scene variations can avoid redundant data acquisition. In such cases, a reconstruction algorithm that can handle measurement data with varying CRs is very valuable. Based on the above discussion, we believe that a simple and easy-to-implement STCI system and a reconstruction algorithm that can handle different CRs are of great significance for the development of STCI. The specific contributions of this work are as follows.

1. Considering the need to balance the encoding efficiency and the simplicity of the imaging system structure, we re-discuss the mathematical model of STCI and adopt a stepwise strategy for spatiotemporal encoding. Through the preprocessing of the mask, we finally only need to introduce a single spatial light modulator (SLM) in the optical system to achieve effective spatiotemporal encoding.
2. By adopting a special structural spatial mask and introducing a hyperparameter module for multi-CR reconstruction in the network, we implemented an STCI reconstruction method that can handle reconstruction with multiple spatiotemporal CRs.
3. Through simulation and optical experiments, we verify that our proposed method can accurately reconstruct the motion and spatial details of high-speed motion scenes when the temporal and spatial compression ratio is 128:1. Similarly, we verify the performance of the reconstruction algorithm under nine spatiotemporal compression ratios.

## 2. Related Work

### 2.1. Compressive Imaging with Different Optical Modulation Devices

Based on the sparsity in different domains, CI has been successfully applied to improve spatial, temporal, or spectral resolution in an imaging system [19,20,21,22]. The CI system mainly consists of a hardware system for data acquisition and back-end algorithms for high-resolution image reconstruction. The high-resolution light modulation device directly affects the complexity and performance of CI systems. In previous research, mainstream high-spatial-resolution light modulation devices include digital micromirror device (DMD) [14], liquid crystal on silicon (LCoS) [15], etc. Although the high resolution SLM increases the complexity of the optical system to some extent, it can realize high-resolution spatial light modulation in different dimensions and can flexibly load modulation masks, so it has been widely used.

With the development of materials and micronano-manufacturing technology, new light modulation devices are used in CI, such as DoE [23] and metasurfaces [24]. These devices can further reduce the complexity of the system and improve the performance of the system, but their application in practical systems is not mature. Single-dimensional CI usually only needs to introduce a single modulation element for single-dimensional encoding. For multidimensional CI, previous research introduces different modulation elements to the system to realize multidimensional encoding [25]. This method can ensure the effectiveness of encoding in different dimensions, but it also increases the complexity of a system.

### 2.2. Typical Reconstruction Algorithms for Spatial or Temporal CI

For the traditional model-based reconstruction algorithm, high-resolution image reconstruction is usually solved as an optimization problem, in which prior information is required. For example, typical model-based algorithms, GAP-TV [26] and TwIST [16], take the total variation (TV) as the prior, while DeSCI [27] takes the low-rank prior into account. The model-based algorithm has strong interpretability and high reconstruction accuracy. However, most traditional model-based reconstruction algorithms adopt iterative methods, which is time-consuming and struggles to meet the real-time requirements of applications. In addition, the model-based algorithm has a strong dependence on prior information, which also limits the performance of the algorithm. DL-based algorithms can be further divided into data-driven and model-driven algorithms. Most data-driven methods are based on the convolution neural network (CNN) [28] or recurrent neural network (RNN) [29]. Model-driven methods fuse the optimization strategy of model-based algorithms into end-to-end networks [18,30], which is more efficient and interpretable. Compared with model-based algorithms, the DL-based method has advantages in reconstruction quality and speed.

## 3. Multiple CR Spatiotemporal Compressive Imaging (Multi-CR STCI)

**Figure 1:**
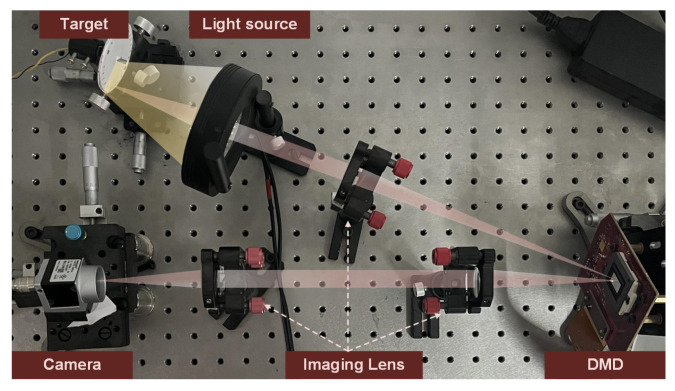
Schematic illustration of the experimental optical configuration for the proposed STCI method.

Figure 1 shows the experimental optical configuration for the proposed STCI method. As shown in the figure, the optical system for STCI is similar to an SCI or TCI system. The imaging system is composed of five main parts: the target, the light source, the imaging lens, the SLM, and the detector array. In the STCI system, the SLM is a critical component that significantly affects system performance. Among the several types of SLM, the DMD offers a compelling combination of spatial resolution, frame rate, and cost-effectiveness. Consequently, we have selected DMD as our spatial light modulator. Light from an object is modulated by patterns displayed on a DMD. The modulated light is then refocused onto a detector array to take measurements. However, the modulation patterns displayed on a DMD and the compressive imaging model are different from the other two types of methods.

### 3.1. Imaging Model and Spatiotemporal Mask Design

In this work, we aim to develop an STCI system that can handle multiple CRs. Thus, we design the modulation patterns separable in the spatial and temporal domains. We call it a stepwise STCI strategy.

**Figure 2:**
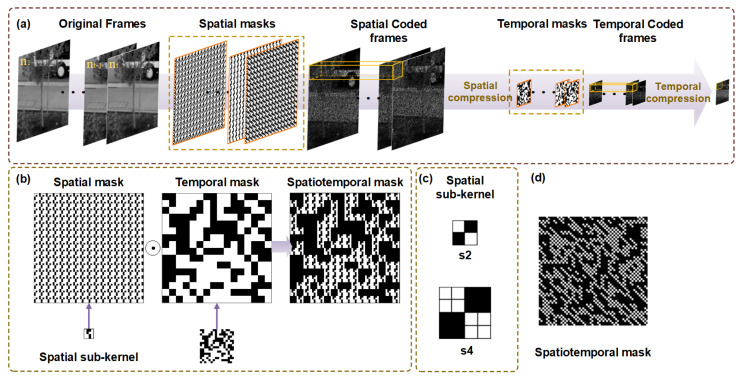
(**a**) Stepwise spatial–temporal encoding and compression model. (**b**) Spatial and temporal mask design and preprocessing for spatiotemporal mask. (**c**) Structure of spatial kernel; s2 is the spatial sub-mask for s=2 and s4 is the spatial sub-mask for s=4. (**d**) One of spatiotemporal masks in this work.

As shown in Figure 2a, the target scenes are initially modulated by spatial masks and compressed in the spatial domain. Then, temporal masks subsequently interact with the spatial compressed frames for temporal modulation. After that, continuous multiple spatiotemporal encoded images are integrated into a single compressed frame within a single exposure period of the camera. The mathematical expression of the STCI imaging model can be expressed as:

(1) $$Y=\sum_{i=1}^{n_{t}}\left(T_{i}⊙\left(B_{add}\left(S_{i}⊙X_{i}\right)\right)\right),$$

in which $X\in R^{n_{h}\times n_{w}\times n_{t}}$ represents target frames with high spatiotemporal resolution. $S\in R^{n_{h}\times n_{w}\times n_{t}}$ indicates the spatial masks. ⊙ is the Hadamard product. We define $B_{add}$ as a block addition operation, which first divides the two-dimensional matrix into non-overlapping blocks with size s×s, and then adds the data in each block to obtain a new matrix with size reduced by *s* times. $B_{add}$ represents the compressed sampling in the spatial domain. $T\in R^{n_{w}/s\times n_{h}/s\times n_{t}}$ is the temporal mask, whose size is the same as that of the spatial compressive images. The subscript *i* is the temporal coordinate of the image sequence. $\sum_{i=1}^{n_{t}}$ represents the temporal compression during one exposure period of the camera. The spatiotemporal CR is composed of temporal CR and spatial CR, which can be expressed as $CR=s^{2}\times n_{t}$.

Stepwise spatial–temporal encoding and compression can achieve the decoupling of spatiotemporal compression to a certain extent, alleviating the difficulty of reconstruction with a large CR. At the same time, considering the complexity of the imaging system, the stepwise strategy requires independent modulation and compression of the spatial and temporal domains, which will inevitably increase the complexity of the system. Therefore, to balance the system complexity and the efficiency of spatiotemporal modulation, we realize equivalent spatiotemporal modulation by preprocessing the spatial and temporal modulation mask. This means that we only need to load a set of spatiotemporal modulation masks to achieve equivalent spatiotemporal modulation as a stepwise method. Observing Equation (1), we can see that the operations contained in the equation are all linear. So we can obtain the following expression by transformation:

(2) $$Y=\sum_{i=1}^{n_{t}}\left(B_{add}\left(C\left(T_{i}\right)⊙S_{i}⊙X_{i}\right)\right),$$

in which we define $C\left(\cdot \right)$ as a matrix expansion operation that enlarges the matrix *s* times by repeating each element in the matrix s×s times. Let $M_{i}=C\left(T_{i}\right)⊙S_{i}$, then Equation (2) can be expressed as Equation (3), which means that we can obtain a set of equivalent spatiotemporal modulation masks by preprocessing the temporal and spatial masks, as shown in Figure 2b.

(3) $$Y=\sum_{i=1}^{n_{t}}\left(B_{add}\left(M_{i}⊙X_{i}\right)\right),$$

Another issue worth noting is that we use a special structural spatial mask to adapt to the multi-CR reconstruction task. As shown in Figure 2c, we chose the quasi-diagonal matrix, in which the upper left and lower right corners of the matrix are 1, and the rest of the matrix is 0, as the spatial sub-kernel. Repeating the spatial sub-kernel in space obtains a spatial mask with the same spatial resolution as the target. This structure can maintain stable original data characteristics with different spatial CRs. Our experimental results also support this point. The temporal masks consist of $n_{t}$ distinct random binary patterns. As discussed previously, we obtain a set of spatiotemporal masks, $M_{i}$, through pre-processing. When we use these masks to modulate the target scene, the DMD sequentially loads the masks. Then, after the modulation, all modulated frames are summed into one measurement frame. Thus, one set of masks is used to make one measurement frame.

**Figure 3:**
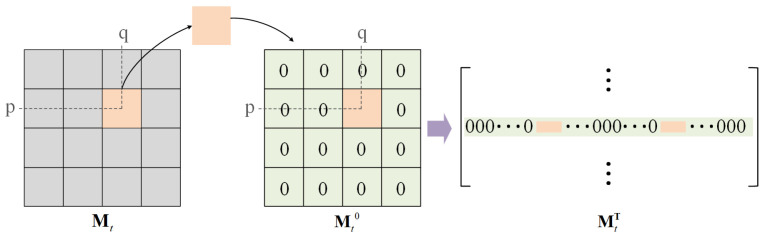
Illustration of transformer for spatial mask.

For further discussion of the imaging model, we rewrite the matrix form in Equation (3) into a vector form:

(4) $$y=Hx=H_{t}H_{s}x,$$

in which $y\in R^{(n_{h}n_{w}/s^{2})\times 1}$ and $x\in R^{(n_{t}n_{h}n_{w})\times 1}$ represent spatiotemporal compressive measurements and original high-resolution frames, respectively. x is composed of $n_{t}$ column vectors $\left\{x_{i},i=1,\cdots ,n_{t}\right\}$ that are concatenated in the column direction, and $x_{i}$ is obtained by reshaping a single high-resolution frame by rows into a column vector. $H\in R^{\left(n_{h}n_{w}/s^{2}\right)\times \left(n_{t}n_{h}n_{w}\right)}$ is the vector form of spatiotemporal masks M in Equation (3). $H_{t}$ and $H_{s}$ represent temporal and spatial masks, respectively. As shown in Figure 3, the $i_{th}$ spatial mask is divided into non-overlapping blocks with a size of s×s, and the block is placed in the corresponding position of a zero matrix of the same size as the spatial mask. The matrix is then expanded into a row vector in a row-first way. Go through all the blocks and concatenate the vectors into a spatial matrix. Finally, $H_{s}$ is obtained by splicing *t* spatial matrices in the row direction. The matrix $H_{t}\in R^{n\times nn_{t}}$ is

(5) $$H_{t}=\left[D_{1},D_{2},\ldots ,D_{n_{t}}\right],$$

with $n=n_{h}n_{w}/s^{2}$ and $D_{i}\in R^{n\times n}$ is obtained by placing the values in the $i_{th}$ temporal mask $T_{i}$ sequentially on the diagonal of the diagonal matrix.

### 3.2. GAP for STCI

In our work, the goal of the algorithm design is to achieve spatiotemporal high-resolution image reconstruction with multiple CRs. We design an unfolding network, STCINet, based on the generalized alternating projection (GAP) [31] algorithm. The optimization problem solved using GAP for STCI is defined as

(6) $$\left(\hat{x},\hat{v}\right)=\underset{x,v}{argmin}\frac{1}{2}{∥x-v∥}_{2}^{2}+\lambda g\left(v\right)\;s.t.\;y=H_{t}\left(H_{s}x\right)$$

Let *k* denote the number of iterations in the GAP. Given $v^{k}$, $x^{\left(k+1\right)}$ can be updated via a Euclidean projection of $v^{k}$ onto the linear manifold $M:y=Hx=H_{t}\left(H_{s}x\right)$:

(7) $$x^{k+1}=v^{k}+H^{T}{\left(HH^{T}\right)}^{-1}\left(y-Hv^{k}\right)),$$

in which $H=H_{t}H_{s}$. Then, a denoiser is used to update $v^{k}$,

(8) $$v^{k+1}=D_{\delta }\left(x^{k+1}\right),$$

where $\delta =\sqrt{\lambda }$ is the standard deviation of the assumed additive white Gaussian noise.

### 3.3. Reconstruction Algorithm

**Figure 4:**
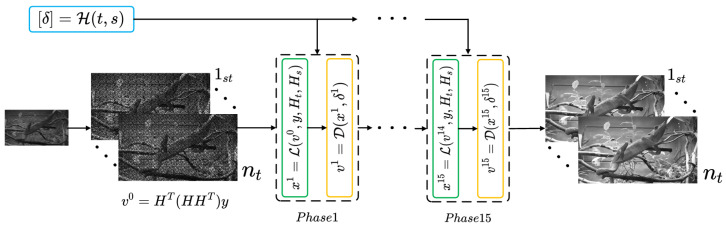
Overall structure of STCINet. It consists of three modules, H (blue box), L (green box), D (yellow box). Module L is for implementing Equation (7), module H is a multi-CR controller and module D is a CNN-based network, which is detailed in Figure 5.

The proposed algorithm is an end-to-end unfolding structure. The core of the unfolding method lies in the combination of the optimization ideas of iterative algorithms with the strong learning capabilities of deep neural networks. By replacing iterations in an iterative method with a network module consisting of several layers, an end-to-end trainable model is constructed. This combination not only preserves the effectiveness of iterative algorithms in solving optimization problems and improves the interpretability of the network, but also takes advantage of deep learning to achieve faster convergence and better generalization ability. As shown in Figure 4, the proposed STCI reconstruction unfolding network is composed of multiple phases stacked together. There are two basic modules in a single phase, L and D, which are designed according to Equation (7) and Equation (10), respectively. Taking into account the balance of computational resources and reconstruction effects, in this work, we set up 15 phases in the reconstruction network. L is a linear operation. According to Equation (7), L updates the target reconstruction $x^{k+1}$ based on the compressed measurement y, the $v^{k}$ output of the previous phase, and the spatial and temporal mask, $H_{t}$, $H_{s}$:

(9) $$x^{k+1}=L\left(v^{k},y,H_{t},H_{s}\right).$$

In module L, $H_{t}$ and $H_{s}$ are fully utilized, making the whole network more explainable.

(10) $$v^{k+1}=D_{\delta }\left(x^{k+1}\right),$$

**Figure 5:**
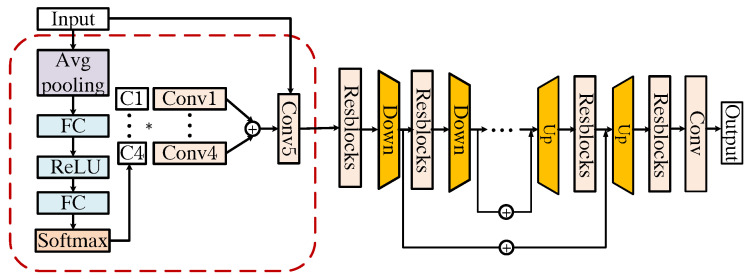
Structure of D(Dy-ResUNet).

The general structure of module D is shown in Figure 5. We design D based on ResUNet [32], in which residual blocks are integrated into a U-Net architecture. In module D, the input is the concatenation of $x^{k+1}$ and a denoise guide map that is filled with the value of δ. Note that in different phases of STCINet, the denoiser parameters, except δ, are the same. More discussion of δ will be presented later. To enhance the performance of the denoiser, we use a dynamic convolution unit (DCU) [33] to generate a kernel for input pre-processing. Therefore, we named module D Dy-ResUNet. The detailed structure of the DCU is shown in the red dotted box in Figure 5. The DCU includes two parts, the self-attention module and a set of convolution layers. The weights of the first *B* convolution layers are calculated from the attention module. Then, the weight of the final convolution layer is the sum of the weights of the first *B* kernels. In DCU, we set B=4 instead of a larger value to avoid hard convergence in network training. The DCU module is followed by a standard encoder–decoder architecture with three down-sample and up-sample stages. Each of them is made up of three residual blocks with 16, 32, and 64 channels. The down-sample operations can reduce the requirement for GPU memory. It is worth mentioning that the module D is designed for single frame input to realize CI reconstruction with multiple CRs, which results in a slightly time-consuming increase for reconstruction.

For multi-CR reconstruction tasks, we introduce the module H into the network. Similarly to [32], the module H is designed to generate a parameter δ for each phase depending on the value of $n_{t},s$, in which *s* represents the spatial CR, and $n_{t}$ represents the temporal CR. There are three fully connected layers in the module H. During the training process, multiple $n_{t}$ and *s* are used to generate the δ values for all *K* phases of the network.

(11) $$\left[\begin{array}{c} \delta \end{array}\right]=H\left(n_{t},s\right).$$

## 4. Simulation Experiments

### 4.1. Network Training

As mentioned in Section 3.3, we have designed a reconstruction network, STCINet, to reconstruct high-spatiotemporal-resolution images from compressive measurements. In this section, we mainly describe the training process, the loss function, and the training settings. We chose DAVIS2017 [34] as the dataset for network training, which contains more than ten thousand continuous images from ninety different high-motion scenes. We cropped these 1080p videos into non-overlapping patches with a size of 8×120×120 for the training process.

We have trained two versions of the reconstruction network for different reconstruction tasks. STCINet-v1 adopts the fixed spatiotemporal CR of 128:1, in which the spatial CR is fixed to s=4 and the temporal CR $n_{t}=8$. The spatiotemporal CR is equal to $s^{2}\times n_{t}$. And STCINet-v2 is trained to solve the multiple CR problem, in which we define the array *s*-bar = [1,2,4], which includes all spatial CRs that can be selected, and the optional $n_{t}$-bar = [2,4,8] as the temporal CR selection array. During every training step, the temporal and spatial CRs are randomly selected from $n_{t}$-bar and *s*-bar. In other words, STCINet-v2 can handle nine spatiotemporal CRs. And it is a pure temporal reconstruction problem when s=1.

We adopt the Adam optimizer to train our model with batch size of 8 and the phase number of the network is set to 15. According to our experiments, more phases are beneficial to the final results and 15 is a proper number considering the trade-off between performance and speed. The initial learning rate is $1\times 10^{-4}$ and decays as the epochs increase until obvious loss drops cannot be observed. In this work, the trained model will be directly used for the reconstruction of compressive measurements captured by optical setup without further fine-tuning.

### 4.2. Results of STCINet_v1

**Figure 6:**
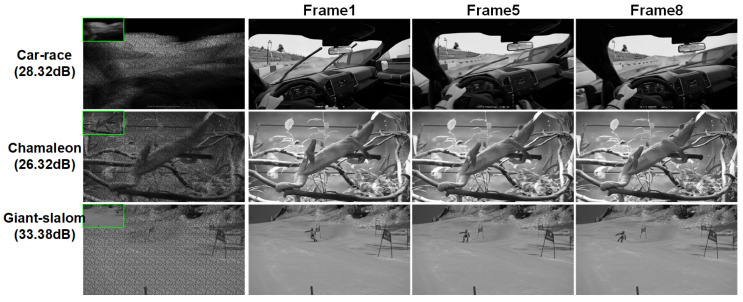
Reconstruction results of STCINet-v1 with CR = 128:1. The first column is the spatiotemporal measurement values and their enlarged images. The second, third and fourth columns are the first, fifth and eighth frames of the reconstructions, respectively.

As described in the above section, STCINet-v1 is trained to tackle the highest spatiotemporal CR, 128:1 ($n_{t}=8,s=4$), which means the sampling rate is lower than 1% in the data acquisition process. We selected 6 videos with a resolution of 1080×1920 to evaluate the model, and the reconstructions of three different scenes are shown in Figure 6. Frames 1, 5 and 8 of the reconstructions are shown to illustrate the performance of the proposed method in the spatial and temporal reconstruction. We can see that STCINet-v1 can recover clear and clean images, whether relatively stationary or high-motion targets, in different motion scenes. We take PSNR (peak signal-to-noise ratio) as the evaluation metric, as shown in Table 1.

### 4.3. Results of STCINet-v2

**Figure 7:**
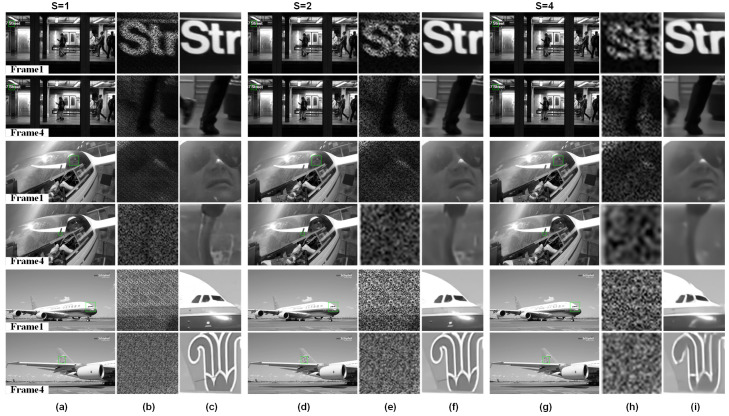
Reconstructions with different spatiotemporal CRs. Temporal CR is fixed to $n_{t}=4$ and spatial CR, *s*, is 1,2,4 respectively. (**a**,**d**,**g**) are reconstructions of different moving targets, in which odd rows are the first frame of reconstructions and even rows are the last frame. (**b**,**e**,**h**) are compressive measurements corresponding to the green box in reconstruction results, and measurements with different spatial CRs are enlarged to the same size for display. (**c**,**f**,**i**) are enlarged images of the box selected area in reconstructions.

**Figure 8:**
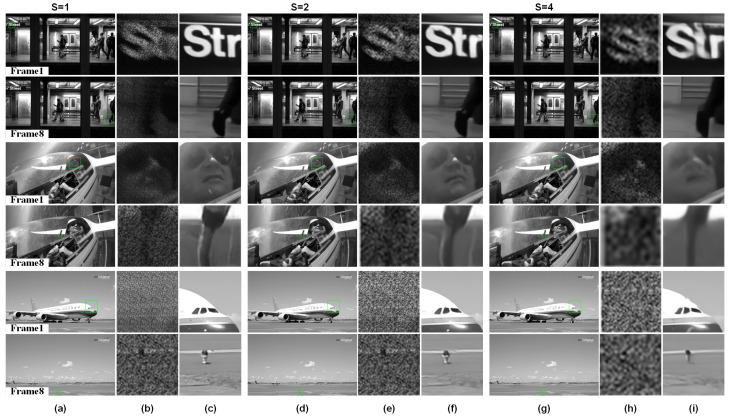
Reconstructions with different spatiotemporal CRs. Temporal CR is fixed to $n_{t}=8$ and spatial CR, *s*, is 1,2,4 respectively. (**a**,**d**,**g**) are reconstructions of different moving targets, in which odd rows are the first frame of reconstructions and even rows are the last frame. (**b**,**e**,**h**) are compressive measurements corresponding to the green box in reconstruction results, and measurements with different spatial CRs are enlarged to the same size for display. (**c**,**f**,**i**) are enlarged images of the box selected area in reconstructions.

STCINet-v2 is designed to reconstruct high-spatiotemporal-resolution images with multiple spatiotemporal CRs. Sequential images with a resolution of $n_{t}\times 1080\times 1920$ are compressed into a single frame with a resolution of (1080/s)×(1920/s). As mentioned above, *s* and $n_{t}$ represent the spatial and temporal CRs, respectively, whose value ranges are [1,2,4] and [2,4,8], respectively. Figure 7 and Figure 8 show the reconstructions when $n_{t}=4$ and $n_{t}=8$, and the spatial CR, *s*, is 1, 2 and 4, respectively.

The first and last frames of the reconstructed images from three different scenes are shown, together with magnified views of the compressed measurements and reconstructions of targets with varying scales and motion patterns in the target scene. For different objects in the moving scene, such as the text in the background with small motion in the subway scene, the tracked helicopter pilot, and the fast sliding plane with large-scale change, STCINet-v2 successfully reconstructs temporal and spatial details from compressed measurements at different CRs. Meanwhile, we can see that with the increase in spatiotemporal CR, the quality of reconstructions decreases to a certain extent, but the temporal and spatial details of the overall image are still well reconstructed. We take PSNR as an evaluation metric, and the PSNR of nine different CRs is calculated and shown in Table 1.

According to the results shown in Table 1, the mean PSNR is 28.99 dB for STCIet-v2 when the spatiotemporal CR is 128:1. Compared with STCINet-v1, there is only a slight decline in the quality of reconstructions, which proves the effectiveness of our method for multi-CR tasks. It can be seen from the results of STCINet-v2 that the quality of reconstructions decreases as the CR increases. Furthermore, by analyzing the influence of increasing temporal CR and increasing spatial CR on reconstructions, we can see that increasing spatial CR will lead to a more obvious decline in the quality of the reconstructions than that of temporal CR. We think the reason is that for high-motion scenes, the sparsity of image sequences in the temporal dimension is higher than the sparsity of the spatial dimension. According to the imaging model, target scenes are modulated by independent temporal and spatial masks, which may fit the redundancy difference in different domains. Moreover, a flexible and efficient network provides the possibility of handling reconstruction tasks with different spatiotemporal CRs.

To further evaluate the performance of STCINet, we conducted VMAF evaluations on 1-sec video sequences with a resolution of 1920 × 1080 and a frame rate of 120 fps [35]. By downsampling the original video to 480 × 270 at 15 fps, we obtained compressed measurements with spatiotemporal CR of 128:1. Our reconstruction algorithm was then used to recover the high-resolution, high-frame-rate video. We evaluated the quality of the reconstructed videos using the VMAF metric under nine different compression ratios for three distinct scenes.

According to Table 2, as the CR increases, the VMAF score of the reconstructed video decreases. When the CR is less than 64:1, the VMAF scores are higher than 80. When the CR is 128:1, the VMAF score of the reconstructed video decreases significantly. We analyze the reasons for this result mainly from two aspects. On the one hand, when we train the network, we use objective evaluation indicators to calculate the loss. Therefore, the reconstruction results will be more inclined to the standards of objective evaluation indicators. In subsequent research, we can consider further improving the algorithm performance by adding subjective evaluation indicators in the training process. On the other hand, there are essential differences between the image degradation factors in compressive imaging and the image degradation factors in video coding, which will also affect the VMAF score.

### 4.4. Temporal Reconstruction

In order to verify the temporal reconstruction capacity of the spatiotemporal reconstruction network, we compared the temporal reconstruction results of STCINet-v2 and other representative temporal compressive imaging reconstruction algorithms, such as GAP-TV, PnP-FFDNet, BIRNAT. The results of other algorithms are obtained by the released code and model weights. In this section, original high-motion scenes are compressed in the temporal domain only, which means *s* is equal to 1 for STCINet-v2. We chose 13 videos with size 8×1080×1920 for testing, which is the same as mentioned in previous simulation experiments. Table 3 shows the average PSNR values of 13 testing scenes, the mean running time, and the maximum GPU memory occupation of different algorithms. GAP-TV and PnP-FFDNet are flexible enough to deal with any temporal CR problem, but they are time-consuming, as those two algorithms adopt the iteration strategy. BIRNAT, which is an end-to-end network based on RNN, produces the best reconstruction result; however, it is memory-consuming that we have to chunk videos into blocks for testing. STCINet-v2 can reconstruct high-resolution videos in 3s with a lower memory cost, and the quality of the reconstruction frames is close to BIRNAT. Moreover, STCINet-v2 can handle multi-CR tasks, while BIRNAT can only handle the specific CR.

**Figure 9:**
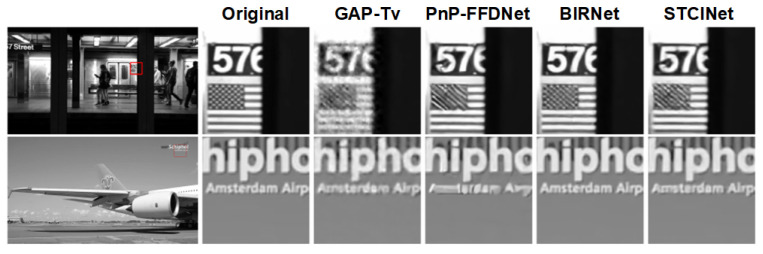
Reconstructions of temporal compressed measurements using different algorithms.

Figure 9 shows one of the temporal reconstruction frames from two different scenes with temporal CR of $n_{t}=8$. Comparing the enlarged details, we can see that GAP-TV and PnP-FFDNet become noisy or over-smooth results. BIRNAT recovers the richest details, and STCINet-v2 performs a high-quality reconstruction that is closer to BIRNAT with a smaller computing source.

### 4.5. Ablation

In this section, two sets of ablation experiments are designed to demonstrate the effectiveness of the modules H and DCU in STCINet-v2. We design an experimental paradigm, in which we remove the two modules from STCINet-v2, respectively, and then the same training strategy is used to train the model. The results of the ablation experiment are compared with the results of STCINet-v2. Table 4 shows the PSNR comparison of the three groups of experiments with nine CRs. w/oH means that the module H is removed from the network. The module H is a multi-CR reconstruction controller, which generates different hyperparameters according to different CRs to adjust the reconstruction process. When module H is removed, the higher CR contributes more to the loss during network training due to the increased difficulty of reconstruction, thus guiding network optimization toward reconstruction tasks of higher CR. On the other hand, at a CR of 32:1, the reconstruction results without module H are better than those with module H, while the reconstruction results at other CRs are inferior to those with module H. We believe this is because the network is unable to adapt to multi-CR tasks and becomes trapped in the optimization of a specific CR. The existence of module H can disperse the attention of the network to different CRs. The final average PSNR shows obvious advantages of H. It can be seen from the table that the introduction of the DCU module has a significant impact on improving the quality of reconstructions with multiple CRs.

## 5. Optical Experiment

### 5.1. Optical Setup

To further verify the effectiveness of the proposed method in practical applications, we built an optical system for verification. Based on the previous analysis, by preprocessing the spatial and temporal masks, we can use only one set of masks to achieve spatiotemporal modulation. Therefore, in the optical system, we only need to introduce one SLM. We choose DMD as the spatial light modulator due to its high frame rate and spatial resolution. The general structure of the optical system is shown in Figure 1. We use a rotating disk driven by an electric motor as the target. Roman numerals and patterns, such as stripes, are printed in circles on the disk. The light source is a customized ring light. We choose DLP6500 as the spatiotemporal modulator. The front imaging system consists of a single lens to image the target onto the DMD, while the back-end imaging system is a 4−f system consisting of two lenses to image the DMD onto the detector. We use Basler ACA720-520 to collect spatiotemporal compressive frames.

Based on the optical system, we have verified the spatiotemporal reconstruction with two CRs. Since the spatial CR is determined by the optical structure, the spatial CR is fixed to s=4 in the optical setup. That is, in the back-end imaging path, the 4×4 pixels in the DMD will converge to a single pixel of the detector. The temporal CR can be adjusted by controlling the frame rate of the DMD and the detector. In the experiment, to guarantee the matching of the compressed measurement and the corresponding masks, we control the detector exposure by the DMD trigger signal. Specifically, DMD sends the trigger signal to the detector when *t* masks are loaded. The detector receives the trigger signal to complete an exposure.

With spatial CR of 16:1 (s=4), the compressed measurement with temporal CR of 4:1 ($n_{t}$ = 4) and 8:1 ($n_{t}$ = 8) were collected, respectively. So the spatiotemporal CRs are 64:1 and 128:1, respectively. In the experiment, we set the frame rate of DMD to 500 fps, while the camera integration time is set to 16 ms (62.5 fps) for spatiotemporal CR of 128:1 and 8 ms (125 fps) for spatiotemporal CR of 64:1. We capture three different objects, the horizontal stripe pattern, the number 9, and the vertical stripe pattern. The spatial resolution of the measurements is 30×30. For two different spatiotemporal CRs, the resolution of reconstructed high-resolution images is 4×120×120 and 8×120×120. We directly use STCINet-v2 trained on the public dataset to reconstruct the high-resolution images from compressed measurements collected by the optical system without additional network fine-tuning.

### 5.2. Optical Results

**Figure 10:**
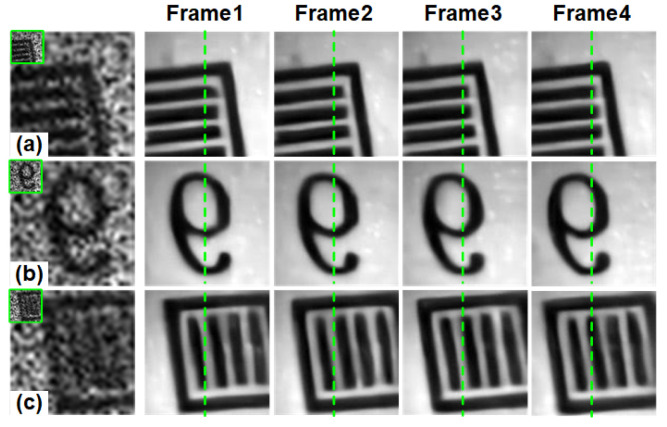
Reconstructions of STCINet-v2 with CR= 64:1, in which $n_{t}=4$, s=4. (**a**–**c**) are spatiotemporal compressive measurements of three different moving targets (green boxes) and their enlarged images. The right side shows the 4-frame spatiotemporal reconstruction image corresponding to the measurement.

**Figure 11:**
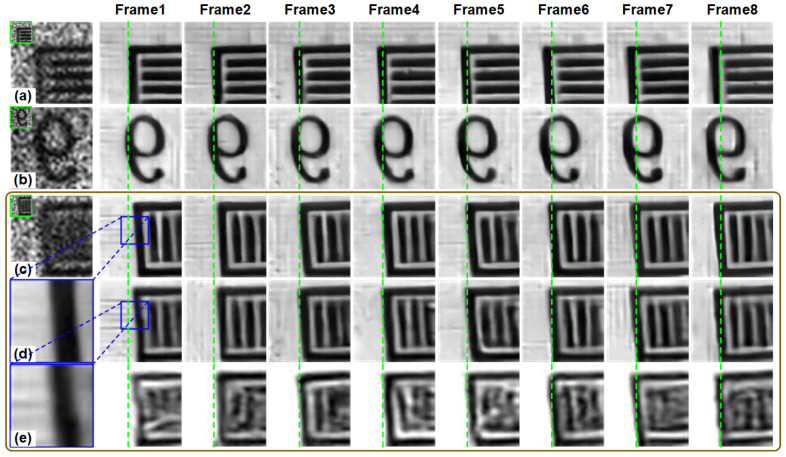
Reconstructions of STCINet-v2 with CR = 128:1, in which $n_{t}=8$, s=4. (**a**–**c**) are spatiotemporal compressive measurements of three different moving targets (green boxes) and their enlarged images. The right is the 8-frame spatiotemporal reconstruction image corresponding to the measurement. The last two rows in the figure are the results of 2 times spatial reconstruction and no spatial reconstruction on the measurement (**c**), respectively. The reconstruction results are interpolated to the same size as that of 4 times spatial reconstruction (row 4). (**d**,**e**) are enlarged images of the blue box in the reconstruction results.

Reconstructions with spatiotemporal CR of 64:1 are shown in Figure 10. From the reconstruction results, we can see that spatial details that cannot be distinguished in the measurements, such as vertical stripes, have been successfully reconstructed. The motion state of the target has also been well reconstructed. Figure 11 shows the reconstructions with spatiotemporal CR of 128:1. Compared with the low spatiotemporal CR of 64:1, the reconstruction quality of the target is still satisfactory even with a higher CR.

To further demonstrate the ability of the algorithm to reconstruct fine details, we performed additional experiments. We reconstruct at different spatial resolutions for compressed measurement with a CR of 128:1, where $n_{t}=8$ and s=4. The fourth and fifth rows of the Figure 11 show the results of reconstructions with 2 times improvement in spatial resolution and without an improvement in spatial resolution, respectively. In the temporal dimension, we reconstructed 8 frames. Specifically, a single measurement frame with the resolution of 30×30 is reconstructed into images with resolutions of 60×60×8 and 30×30×8, respectively. For reconstructions without improvement in spatial resolution, the fine spatial details of the target are indistinguishable. The 2 times reconstruction shows a marked improvement in spatial resolution, but the boundaries of the stripes remain somewhat fuzzy. However, 4 times reconstructions yield a much more complete recovery of the target’s spatial details. These results suggest that spatiotemporal reconstruction is capable of effectively recovering detailed information of moving targets.

### 5.3. Discussions

The optical experiments validated the effectiveness and feasibility of the STCI method. Experimental results demonstrate that the imaging system can achieve up to a 4 times improvement in spatial resolution and an 8 times enhancement in temporal resolution simultaneously using the STCI. Note that the spatial and temporal resolutions of DMD restrict the system’s resolutions. However, it is not uncommon for a DMD to have spatial resolution greater than 1024×1024 and frame rate greater than 10 K fps, which can meet the requirements in most applications.

In addition, dynamic range is a critical performance metric for imaging systems. We discuss the dynamic range in STCI from two perspectives. The first is about compressive measurements. If we assume that a compressive measurement frame accumulates temporal and spatial original scene pixels, then the measurements can easily exceed the dynamic range of a detector. To address the issue, in the simulated experiments, we multiply the template by a factor $1/s^{2}B$, where *B* is the number of frames compressed into one measurement frame, and *s* represents that s×s pixels are spatially compressed into one pixel of a measurement frame. This ensures that the measurement frame is still in the dynamic range of a detector. The second is about the dynamic range in terms of each detectable object frame. Although the STCI method improves the resolution of the system, it introduces a reduction in the dynamic range of the original object frames. In STCI, we optically compress the light from the s×s×B object pixels into one STCI measurement. Thus, the dynamic range of a detector pixel is dispersed into *B* object frames. The dynamic range of the detectable object frames is reduced. This is consistent with what appears in high-speed cameras. As the speed increases, the object light for each frame is reduced. This makes the noise more noticeable. However, in STCI, the reconstruction network has been designed and trained to deal with noise. Hence, the back-end algorithm can compensate for the loss of dynamic range during reconstruction, ensuring that no significant dynamic range degradation is observed in the final results.

## 6. Conclusions

In this paper, we present a spatiotemporal compressive imaging (STCI) method. To balance the encoding efficiency and system simplicity in STCI, we revisited the mathematical model of STCI and introduced a stepwise strategy for spatiotemporal encoding. By carefully preprocessing the mask, we demonstrate that effective spatiotemporal encoding can be achieved with a single spatial light modulator (SLM), significantly simplifying the optical system. Furthermore, we developed a novel deep learning-based reconstruction method that incorporates a hyperparameter module to enable reconstruction across a range of spatiotemporal compression ratio (CRs). Through extensive simulations and experimental validations, we verified the efficacy of our approach in accurately reconstructing high-speed motion scenes with a spatiotemporal compression ratio of 128:1. Importantly, our method demonstrated robust performance across nine different spatiotemporal CRs, highlighting its versatility and adaptability. This work explores the development of more efficient and practical STCI systems with enhanced capabilities for capturing dynamic scenes. In our future work, we will further explore the improvement of image quality in spatiotemporal compressive imaging from both the perspectives of imaging models and imaging algorithms.

## Figures and Tables

**Table 1:** PSNR (dB) using STCINet-v2/STCINet-v1 with different compression ratios.

$n_{t}/s/\mathrm{CR}$	2/1/2:1	2/2/8:1	2/4/32:1	4/1/4:1	4/2/16:1	4/4/64:1	8/1/8:1	8/2/32:1	8/4/128:1
Subway	46.54	38.75	31.63	43.63	36.74	30.68	39.75	33.53	28.33/28.56
Aerobatics	36.79	34.22	30.97	35.25	33.04	30.20	33.69	31.38	28.48/28.79
Plane	42.26	34.38	29.10	40.82	34.30	29.29	38.89	34.15	29.98/30.16
Chameleon	42.72	34.45	28.77	39.20	31.99	27.96	35.05	29.19	25.82/26.09
Car-race	41.15	34.54	27.87	39.72	33.72	28.73	38.56	33.37	27.91/28.40
skiing	44.54	39.09	35.25	41.99	37.57	39.12	35.84	29.89	33.44/33.69
Avg.	42.33	35.91	30.60	40.10	34.56	31.00	36.96	31.92	28.99/29.28

**Table 2:** VMAF with different compression ratios.

$n_{t}/s/\mathrm{CR}$	2/1/2:1	2/2/8:1	2/4/32:1	4/1/4:1	4/2/16:1	4/4/64:1	8/1/8:1	8/2/32:1	8/4/128:1
Beauty	91.94	89.34	81.63	90.15	86.41	77.88	86.81	80.96	71.56
Bosphours	99.93	99.90	89.33	99.92	97.69	79.31	99.41	89.43	66.15
Honeybee	91.94	89.34	81.63	90.15	86.41	77.88	86.81	80.96	78.41
Avg.	94.60	92.86	84.19	93.40	90.17	78.35	91.01	83.78	72.04

**Table 3:** Comparison of different TCI algorithms (PSNR: dB, running time: s, memory: MB).

Algorithms	Average PSNR	Running Time	Memory
GAP-TV	32.01	39.5	-
PnP-FFDNet	35.93	29.8	9017
BIRNAT	37.9	3.8	>24,000
Ours	37.14	2.9	3199

**Table 4:** PSNR (dB) in ablation experiments.

$n_{t}/s/\mathrm{CR}$	2/1/2:1	2/2/8:1	2/4/32:1	4/1/4:1	4/2/16:1	4/4/64:1	8/1/8:1	8/2/32:1	8/4/128:1	Avg.
**STCINet**	**42.33**	**35.91**	30.60	**40.10**	**34.56**	**31.00**	**36.96**	31.92	**28.99**	**34.68**
**w/o** H	38.67	34.72	**30.66**	37.41	33.53	30.07	35.39	**32.03**	28.86	33.48
**w/o DCU**	38.41	34.28	30.56	36.89	33.19	29.87	34.94	31.75	28.70	33.29

## Data Availability

Data underlying the results presented in this paper are not publicly available at this time but may be obtained from the authors upon reasonable request.
